# Impact of Implementing an Outpatient Antimicrobial Stewardship Program on Urinary Tract Infection Management in a Private Hospital in Costa Rica

**DOI:** 10.7759/cureus.93646

**Published:** 2025-10-01

**Authors:** Carolina Rojas-Chinchilla, José Pablo Díaz-Madriz, José Chaverri-Fernández, Sebastián Arguedas-Chacón, Ariana Araya-Mena, Guillermo Fernández-Aguilar, Gabriel Muñoz-Gutiérrez, Esteban Zavaleta-Monestel

**Affiliations:** 1 Antimicrobial Stewardship Program, Hospital Clínica Bíblica, San José, CRI; 2 Pharmacology, Toxicology, and Drug Dependence, Universidad de Costa Rica, San José, CRI; 3 Research, Hospital Clínica Bíblica, San José, CRI; 4 Faculty of Pharmacy, Universidad de Costa Rica, San José, CRI; 5 Clinical Medicine, Hospital Clínica Bíblica, San José, CRI; 6 Infectious Diseases, Hospital Clínica Bíblica, San José, CRI; 7 Pharmacy, Hospital Clínica Bíblica, San José, CRI

**Keywords:** antimicrobial stewardship program, clinical pharmacist, clinical practice guidelines, outpatient, urinary tract infection

## Abstract

Background: Antimicrobial stewardship programs (AMS) improve antibiotic use in both hospital and outpatient (OP) settings. While AMS initiatives in Latin America have focused mainly on inpatient care, OP-specific interventions remain limited. Hospital Clínica Bíblica (HCB) had an AMS program for hospital settings but lacked structured OP activities.

Objectives: To evaluate the implementation of an AMS-OP in a private hospital in Costa Rica, its impact on antibiotic prescribing patterns for urinary tract infections (UTIs), and its feasibility for broader application in the Latin American context.

Methods: A retrospective observational study with a pre- and post-intervention design was conducted. The analysis compared OP antibiotic prescribing for UTIs during the pre-AMS-OP period (July 2021-March 2022) and the post-AMS-OP period (July-December 2022). The AMS-OP was implemented from April to June 2022. Baseline compliance with CDC AMS-OP elements was assessed. A total of 269 OP UTI cases were analyzed, focusing on optimal antibiotic selection, physician adherence to clinical guidelines, and clinical outcomes, including recurrence and relapse.

Results: UTIs accounted for 163 (40.1%) of 407 OP antibiotic prescriptions. Pre-AMS-OP, 255 (62.7%) of the antibiotics prescribed belonged to the WHO Watch category. Post-AMS-OP, CDC compliance increased from 28.6% to 85.7%. Optimal antibiotic selection improved from 143 (53.8%) to 126 (95.2%) cases (p < 0.001). The optimal use of ciprofloxacin and levofloxacin improved by 31.3% (p = 0.028) and 60.0% (p = 0.027), respectively. Nitrofurantoin and fosfomycin use increased, while trimethoprim-sulfamethoxazole (TMP-SMX) and fluoroquinolones decreased. Physician adherence scores rose from 46.2 to 90.0. UTI recurrence decreased to 15 cases (11.9%, p = 0.005), and relapse rates fell to 3.1% (p = 0.07).

Conclusions: Implementing AMS-OP based on CDC elements significantly improved antibiotic selection, physician guideline adherence, and clinical outcomes for UTIs in the OP setting. This strategy appears feasible and beneficial for OP care in Latin America.

## Introduction

Antimicrobial resistance (AMR) is a growing global public health threat. The World Health Organization (WHO) identifies AMR as one of the top ten global health challenges, attributing approximately 33,000 deaths annually in Europe alone, with the global toll likely much higher. The overuse and misuse of antimicrobials accelerate the emergence of resistant pathogens and facilitate bacterial adaptation, complicating infection management and treatment outcomes [1,2]. 

To address this crisis, the WHO published the Global Action Plan on AMR in 2015, outlining five strategic objectives: increasing awareness and understanding of AMR; strengthening surveillance and research; reducing the incidence of infection through effective hygiene; optimizing antimicrobial use; and promoting investment in new medicines, diagnostics, vaccines, and other interventions [3]. 

Among these strategies, implementing antimicrobial stewardship programs (AMS) is a key recommendation. AMS aims to improve patient outcomes, reduce AMR, and prevent healthcare-associated infections by promoting the appropriate use of antimicrobials. While AMS programs are well established in hospital settings, AMS-outpatient (OP) initiatives, though less common, have shown promise in enhancing antibiotic selection and reducing inappropriate prescriptions [4]. 

The CDC proposes four core elements for AMS-OP implementation: commitment; action through policy and practice; tracking and reporting; and education and expertise support [5]. AMS-OP typically targets high-prevalence infections such as bacterial pneumonia, skin and soft tissue infections, and urinary tract infections (UTIs). However, there is limited evidence on the implementation and effectiveness of AMS-OP programs in Latin America [6]. 

Hospital Clínica Bíblica (HCB) in San José, Costa Rica, has implemented an AMS program primarily focused on inpatient care. This initiative has improved antibiotic consumption patterns for agents such as cefazolin, ceftriaxone, and levofloxacin, influencing prescribing behaviors in surgical and intensive care units [7-9]. Nevertheless, structured AMS activities for OP prescriptions are lacking. An initial analysis at HCB pharmacies revealed that most OP antibiotic prescriptions were for UTIs. 

Inappropriate antibiotic prescribing is recognized by the CDC as a leading modifiable driver of AMR [5,10]. Given the complex resistance mechanisms of uropathogens, managing UTIs remains a clinical challenge, underscoring the importance of AMS-OP in this context [11]. 

This study aims to evaluate the implementation of an AMS-OP at HCB, assess its impact on OP prescribing patterns and clinical outcomes for UTIs, and explore its feasibility for broader application in Latin America. 

## Materials and methods

Study design 

This was a retrospective observational study using a pre- and post-intervention design. The analysis evaluated the impact of implementing an AMS-OP at Hospital Clínica Bíblica, San José, Costa Rica. Data were collected from electronic health records and pharmacy dispensing systems for three periods: pre-AMS-OP (July 2021-March 2022), implementation (April-June 2022), and post-AMS-OP (July-December 2022). Ethical approval was obtained from the Scientific Ethics Committee of the University of Costa Rica (CEC-284-2022). 

AMS-OP implementation and evaluation 

To characterize baseline OP antibiotic prescribing patterns, prescriptions dispensed at HCB pharmacies from January to March in both 2021 and 2022 were reviewed. Each prescription was classified using the anatomical therapeutic chemical (ATC) classification system, infection type, source of prescription, and the WHO Access, Watch, and Reserve (AWaRe) categorization [12]. Baseline compliance with the CDC’s AMS-OP core elements was assessed using the official CDC evaluation framework [5]. 

Based on these findings, an AMS-OP action plan was developed and implemented. This included the creation of clinical guidelines, voluntary training sessions for prescribers, the distribution of educational materials, and the establishment of monitoring protocols, all of which were aligned with the CDC’s recommended core elements. The emergency department was selected as the primary intervention site due to its high volume of infection-related visits and availability of detailed prescription records. 

Post-implementation assessment 

Following the AMS-OP implementation, compliance with CDC core elements was reassessed. The program’s impact was evaluated by comparing prescribing patterns between the pre- and post-implementation periods. Key outcomes included the rate of optimal empirical antibiotic selection, adherence to clinical guidelines, and clinical outcomes related to UTIs. 

Clinical outcomes and definitions 

Clinical outcomes focused on recurrence and relapse of UTIs. Recurrence was defined as two or more UTIs within six months or three or more within one year. Relapse was defined as a recurrent UTI occurring within two weeks of treatment completion, caused by the same pathogen [13]. 

Collected variables included patient demographics (age, sex, comorbidities), urine culture results, risk factors, and parameters related to empirical antibiotic selection, dosage, and treatment duration. These parameters were evaluated based on a clinical guideline developed from evidence-based recommendations from the Infectious Diseases Society of America (IDSA), the European Association of Urology (EAU), and the Sanford Guide (Antimicrobial Therapy, Inc., VA, USA. 

Data sources 

Data were extracted from electronic hospital records and pharmacy dispensing databases. Variables collected included infection type, prescribing physician, prescribed antibiotic, route of administration, treatment duration, and dosage. 

Inclusion and exclusion criteria 

Inclusion criteria comprised patients aged ≥18 years who received empirical OP treatment for UTIs in the emergency department. Exclusion criteria included individuals under 18, pregnant women, patients requiring hospitalization, and those with incomplete medical records. 

Statistical analysis 

Descriptive statistics were calculated for each study period. Categorical variables were compared using the chi-square or Fisher’s exact tests, while continuous variables were analyzed using independent-samples t-tests or Wilcoxon rank-sum tests. Odds ratios (ORs) with 95% confidence intervals (CIs) were computed to assess associations. A two-tailed p-value < 0.05 was considered statistically significant. Data analysis was performed using Microsoft Excel 2019 (Microsoft Corporation, Redmond, Washington, United States), IBM SPSS Statistics for Windows, Version 21 (Released 2012; IBM Corp., Armonk, New York, United States), and R Studio version 4.3.0 (RStudio: Integrated Development Environment for R. Boston, MA). 

## Results

A total of 407 OP antibiotic prescriptions dispensed at HCB pharmacies between January and March of 2021 and 2022 were analyzed. UTIs accounted for 163 (40.1%) of prescriptions, making them the most frequent indication, followed by gastrointestinal conditions (n = 86, 21.1%), respiratory infections (n = 71, 17.4%), skin and soft tissue infections (n = 55, 13.5%), ocular, oral, and ear infections (n = 17, 4.2%), and gynecological infections (n = 15, 3.7%). 

**Figure 1:**
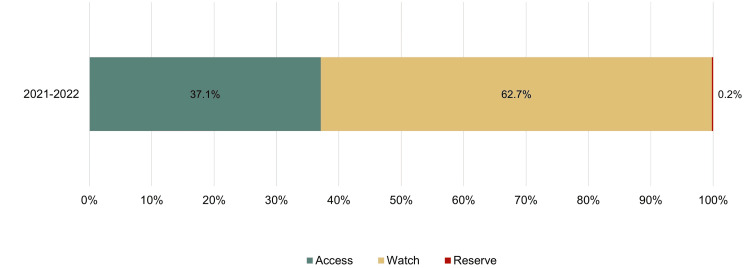
Percentage distribution of outpatient antibiotic use according to the WHO’s AWaRe classification. AWaRe: Access, Watch, and Reserve

Among antibiotics prescribed for UTIs, fluoroquinolones were the most frequently used (n = 65, 39.9%), followed by fosfomycin (n = 26, 16.0%), third-generation cephalosporins (n = 17, 10.4%), nitrofuran derivatives (n = 13, 8.0%), and trimethoprim-sulfamethoxazole (TMP-SMX) (n = 8, 4.9%). The remaining 20.9% (n = 34) corresponded to other antibiotic classes. According to the WHO AWaRe classification, 255 (62.7%) antibiotics were classified as Watch, 151 (37.1%) as Access, and one (0.2%) as Reserve, indicating suboptimal alignment with WHO recommendations, particularly with respect to Access-category utilization (Figure 1). 

AMS-OP compliance and implementation 

Prior to AMS-OP implementation, compliance with CDC-recommended core elements was 28.6%. Following the intervention, compliance increased to 85.7%. The greatest improvements were observed in prescriber education and in monitoring and reporting activities. These improvements were driven by the development of clinical guidelines, in-person training sessions, the dissemination of educational materials, and the incorporation of digital tools to track compliance indicators (Table 1).

**Table 1:** Implementation of HCB’s AMS-OP based on CDC standards. AMS-OP: outpatient antimicrobial stewardship; HCB: Hospital Clínica Bíblica

CDC Core Element	Pre–AMS-OP Context	Activities Implemented During AMS-OP	Pending Actions
Commitment	- AMS-HCB established in 2015. - Supported by hospital leadership, with dedicated staff and regular reporting of intra-hospital activities.	- Prescribers informed of institutional antimicrobial use policy.- Visual materials (posters) placed in offices and the hospital bulletin to demonstrate institutional commitment.	–
Action and Policy	- No legal restriction on outpatient antibiotic dispensing. - Existing hospital clinical guidelines include outpatient prescriptions. - Pharmacists support available via the hospital contact center.	- Clinical guidelines for UTIs reviewed and updated. - Promotion of watchful waiting and justification of first-line deviations. - Dissemination of PRO-AMB contact for clinical support. -Multichannel access to clinical guidelines, antimicrobial resources, and local resistance data.	- Require explicit written justification in medical records for non-recommended antibiotic prescriptions.
Monitoring and Reporting	- No established outpatient antibiotic use indicators.	- Creation of Power BI (Microsoft Corporation, Redmond, Washington, United States) monitoring dashboards on compliance by infection type, prescriber, and commonly used antibiotics. - In-person feedback sessions on clinical guideline adherence. - Sharing prescriber performance reports based on guideline compliance. - Establishment of individual and institutional performance targets.	- Monitor the percentage of visits resulting in antibiotic prescriptions.
Education	- No outpatient antimicrobial training available for prescribers.	- In-person training on clinical guidelines and resistance trends. - Educational posters developed for physicians and patients. - Implementation of the Sanford Guide mobile app (Antimicrobial Therapy, Inc., VA, USA).	- Develop a continuous education strategy for outpatient antimicrobial use.

Optimal empirical antibiotic selection 

A total of 269 UTI cases were included in the evaluation of empirical treatment appropriateness, 143 during the pre-AMS-OP period and 126 during the post-AMS-OP period. The proportion of cases with optimal empirical antibiotic selection increased significantly, from 76 of 138 cases (55.1%) in the pre-AMS-OP period to 120 of 126 cases (95.2%) in the post-AMS-OP period (p < 0.001) (Table 2)

**Table 2:** Comparison of optimal antibiotic selection for empirical UTI treatment before and after AMS-OP implementation. ▲: Increase; ▼: Decrease; *: magnitude of change (%); pre-AMS-OP: pre-outpatient antimicrobial stewardship implementation; post-AMS-OP: post-outpatient antimicrobial stewardship implementation; UTI: urinary tract infection; TMP-SMX: trimethoprim-sulfamethoxazole

Antibiotic	Pre-AMS-OP N = 138, (%)	Pre-AMS-OP Optimal n = 76, (%)	Post-AMS-OP N = 126, (%)	Post-AMS-OP optimal n = 120, (%)	Optimal Selection^*^	Prescription Rate*	P-value
Amoxicillin- clavulanate	1 (0.7)	1 (100.0)	19 (15.1)	19 (100.0)	0 .0	▲14.4	-
Cefixime	23 (16.7)	22 (95.7)	13 (10.3)	13 (100.0)	▲4.3	▼-6.4	1.000
Ceftriaxone	5 (3.6)	3 (60.0)	4 (3.2)	4 (100.0)	▲40.0	▼-0.4	0.440
Cefuroxime	9 (6.5)	9 (100.0)	12 (9.5)	12 (100.0)	0.0	▲3.0	-
Ciprofloxacin	48 (34.8)	24 (50.0)	16 (12.7)	13 (81.3)	▲31.3	▼-22.1	0.028
Ertapenem	2 (1.4)	1 (50.0)	1 (0.8)	1 (100.0)	▲50.0	▼-0.6	1.000
Fosfomycin	9 (6.5)	9 (100.0)	29 (23.0)	28 (96.6)	▼-3.4	▲16.5	0.572
Levofloxacin	5 (3.6)	2 (40.0)	9 (7.1)	9 (100.0)	▲60.0	▲3.5	0.027
Nitrofurantoin	5 (3.6)	5 (100.0)	21 (16.7)	21 (100.0)	0.0	▲13.1	-
TMP-SMX	31 (22.5)	0 (0.0)	2 (1.6)	0 (0.0)	0.0	▼-20.9	-

Specifically, ciprofloxacin prescribing improved by 31.3% (OR = 0.23, 95% CI: 0.058-0.915; p = 0.028), and levofloxacin by 60.0% (p = 0.027). Use of nitrofurantoin and fosfomycin increased by 13.1% (n = 21) and 16.5% (n = 28), respectively, while TMP-SMX and overall fluoroquinolone use decreased by 20.9% and 18.6%, respectively. 

When stratified by infection type, optimal treatment for uncomplicated cystitis improved by 56.0% (p < 0.001), and for complicated cystitis by 9.6% (p = 0.05) (Table 3). 

**Table 3:** Comparison of the optimal selection of empirical treatment according to diagnosis between the pre-AMS-OP and post-AMS-OP periods. ▲: Increase; *: magnitude of change (%); pre-AMS-OP: pre-outpatient antimicrobial stewardship implementation; post-AMS-OP: post-outpatient antimicrobial stewardship implementation

Diagnosis	Pre–AMS-OP (n optimal / N)	% Optimal	Post–AMS-OP (n optimal / N)	% Optimal	Change in % Optimal	P-value
Uncomplicated cystitis	36 / 96	37.5%	58 / 62	93.5%	▲56.0	0.001
Complicated cystitis	31 / 35	88.6%	55 / 56	98.2%	▲9.6	0.050
Pyelonephritis (low resistance risk)	10 / 12	83.3%	7 / 7	100.0%	▲16.7	0.509
Asymptomatic bacteriuria	0 / 0	–	0 / 1	0.0%	0.0	–

Physician adherence to clinical guidelines 

The average physician adherence score improved from 46.2 pre-AMS-OP to 90.0 post-AMS-OP (p < 0.001). Adherence was assessed using a 100-point checklist, where each correctly followed guideline item contributed one point. In the post-AMS-OP period, over 80% of physicians scored between 85 and 100 points, demonstrating substantial improvement in compliance with clinical practice guidelines. 

Clinical outcomes 

Table 4 presents the demographic and clinical characteristics, treatment regimens, and microbiological findings of patients who experienced a relapse of UTI in both the pre- and post-AMS-OP periods. During the pre-AMS-OP period, UTI recurrence occurred in 36 of 143 patients (25.2%), and relapse in 12 (8.4%). The patients had an average age of 58.6 years, with women constituting 58.3% of the group. Among the identified risk factors were recent hospitalization (within the previous 90 days) in one patient and catheter use in two patients. Regarding the initial empirical treatment, optimal UTI treatment was administered to 50.0% (n = 6) of patients, the correct dosage was given to 75.0% (n = 9), and 41.6% (n = 5) received treatment for the appropriate duration. Urine culture tests were conducted on all patients, revealing *E. coli *as the predominant in 58.3% (n = 7) of cases, followed by *K. pneumoniae* in 25.0% (n = 3). Extended-spectrum beta-lactamase (ESBL) production was detected in 41.7% (n = 5) of the isolates.

**Table 4:** Demographic characteristics and treatment of patients with UTI relapse. –: not applicable or not present; SD: standard deviation; ESBL: extended-spectrum beta-lactamase; pre-AMS-OP: pre-outpatient antimicrobial stewardship implementation; post-AMS-OP: post-outpatient antimicrobial stewardship implementation; UTI: urinary tract infection Optimal selection, dose, and duration were defined according to institutional clinical guidelines.

Variable	Pre-AMS-OP (n = 12)	Post-AMS-OP (n = 4)
Age	58.6 ±17.1	54.3 ±17.7
Gender		
Male	5 (41.7%)	2 (50.0%)
Female	7 (58.3%)	2 (50.0%)
Morbilities		
Hypertension	6 (50.0%)	2 (50.0%)
Diabetes	3 (25.0%)	-
Arrhythmias	-	1 (25.0%)
Dyslipidemia	3 (25.0%)	1 (25.0%)
Benign prostatic hyperplasia	1 (8.3%)	1 (25.0%)
Nephrolithiasis	2 (16.7%)	-
Pulmonary embolism	1 (8.3%)	-
Diverticulitis	1 (8.3%)	-
Hypothyroidism	1 (8.3%)	-
Risk Factors		
Prior hospitalization within 90 days	1 (8.3%)	-
Catheter	2 (16.7%)	1 (25.0%)
Antibiotic Selection	12 (100.0%)	4 (100.0%)
Amoxicillin- clavulanic acid	2 (16.7%)	-
Ceftriaxone	2 (16.7%)	1 (25.0%)
Cefuroxime	1 (8.3%)	-
Ciprofloxacin	2 (16.7%)	-
Ertapenem	3 (25.0%)	-
Fosfomycin	-	2 (50.0%)
Gentamicin	1 (8.3%)	-
TMP-SMX	1 (8.3%)	1 (25.0%)
Optimal Dose	9 (75.0%)	4 (100.0%)
Optimal Duration	5 (41.6%)	3 (75.0%)
Bacteria		
Escherichia coli	7 (58.3%)	4 (100.0%)
Klebsiella pneumoniae	3 (25.0%)	-
Streptococcus parasanguinis	1 (8.3%)	-
Proteus vulgaris	1 (8.3%)	-
ESBL	5 (41.7%)	1 (25.0%)
Quinolone resistant	2 (16.7%)	-

Post-intervention, recurrence decreased to 15 of 126 patients (11.9%) (p = 0.005), and relapse to four (3.1%) (p = 0.07). The average age of patients was slightly lower, at 54.3 years, with an equal distribution between male and female patients. Among the patients, only one was reported to have a catheter, and none had recent hospitalizations. The initial empirical treatment showed improvement, with 75.0% (n = 3) of patients receiving UTI treatment for optimal duration and all patients (100.0%, n = 4) receiving the correct dosage. Urine culture tests were conducted on all patients, with *E. coli *detected in all of the isolates. During this period, ESBL resistance genes were identified in only one isolate.

AWaRe classification of antibiotic use in UTIs 

**Figure 2:**
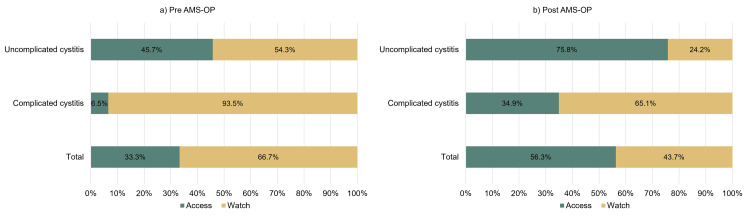
Percentage of antibiotic use according to the WHO AWaRe classification by diagnosis. a: pre-AMS-OP; b: post-AMS-OP AMS-OP: outpatient antimicrobial stewardship; AWaRe: Access, Watch, and Reserve

Following AMS-OP implementation, antibiotic prescribing for UTIs showed notable improvements in alignment with WHO targets. Use of Access-category antibiotics for uncomplicated cystitis increased from 45.7% to 75.8%. For complicated cystitis, Watch-category use decreased from 93.5% to 65.1%. Overall, Access-category antibiotic use for UTIs increased by 23.0% post-implementation (Figures 2a, 2b). 

## Discussion

The retrospective observational design with pre- and post-intervention comparison allowed assessment of prescribing behaviors and clinical outcomes associated with AMS-OP implementation in a real-world OP setting.

The baseline analysis of antibiotic prescriptions at HCB revealed that UTIs were the most common indication, accounting for 163 (40.1%) of OP prescriptions. This aligns with global data indicating that UTIs are the most prevalent OP infection [14]. Worldwide, an estimated 150 million individuals develop a UTI annually, representing approximately 0.7% of all OP visits and 15% of OP antibiotic prescriptions in the United States [15]. The burden appears to be even greater in Latin America, consistent with our findings [16]. 

Respiratory and skin and soft tissue infections also ranked among the most common diagnoses after UTIs, mirroring global trends [6,17]. However, given that many gastrointestinal infections do not require antibiotic therapy, focusing stewardship efforts on UTIs and other high-frequency conditions with clearer indications for antibiotics represents a more rational approach in OP settings [17]. 

Fluoroquinolones and third-generation cephalosporins were among the most frequently dispensed antibiotics, despite their high epidemiological impact and associated risks. These agents should not be first-line options in most OP infections [17,18]. Fluoroquinolones, in particular, are associated with serious adverse events such as tendinopathy, peripheral neuropathy, and central nervous system toxicity and should be reserved for use only when no safer alternatives are available [19]. Nevertheless, they continue to be widely prescribed in ambulatory care. 

The overuse of broad-spectrum antibiotics in OP settings becomes more evident when prescribing patterns are analyzed using the WHO AWaRe classification. While the WHO recommends that antibiotics from the Access group comprise at least 60% of total antibiotic use, only 37.1% of prescriptions in our baseline data met this target, highlighting the need for targeted interventions, such as AMS-OP [18]. 

To address these concerns, a structured action plan was implemented at HCB, aligned with the CDC’s core AMS elements. Following implementation, 12 of the 14 elements were fulfilled, demonstrating the feasibility of deploying such standards in Costa Rica and potentially across the Latin American region. Adherence to these standards has been linked to improved antimicrobial prescribing [4,20,21]. However, certain CDC-recommended elements, such as monitoring the percentage of total visits resulting in antibiotic prescriptions and requiring justification in medical records for non-guideline-based prescriptions, could not be implemented. These strategies are known to enhance prescribing quality [20,21], but are difficult to enforce in private healthcare settings lacking prescription restrictions. Broader implementation may depend on national initiatives, such as a centralized digital antibiotic prescription platform [18]. 

To assess the AMS-OP impact, efforts were focused on educating emergency department prescribers and monitoring their practices. Emergency departments are ideal AMS-OP targets due to their high patient volumes, transitional role between inpatient and OP care, and frequent initiation of empirical therapy. The CDC recommends their inclusion in stewardship programs [22]. 

Empirical antibiotic selection improved by 41.4% (p < 0.001) after AMS-OP implementation, consistent with outcomes from similar interventions in emergency departments [6,23]. Key strategies included distributing prescribing reports, displaying educational materials in clinical areas, implementing evidence-based guidelines, and providing individual feedback on prescribing behavior [18,20,24]. 

As shown in Table 2, improved prescribing translated into more appropriate use of antibiotics commonly used to treat UTIs [6,22,23]. Notably, ciprofloxacin and levofloxacin prescribing improved by 31.3% (p = 0.028) and 60.0% (p = 0.027), respectively. Simultaneously, the use of TMP-SMX and fluoroquinolones decreased, while nitrofurantoin and fosfomycin, first-line agents, were prescribed more frequently. The reduction in TMP-SMX use is especially relevant, given high resistance rates in Costa Rica and at HCB [18,25]. These improvements likely contributed to enhanced infection management, reduced resistance, and improved patient safety [7,26]. 

Treatment optimization was particularly evident in cases of uncomplicated cystitis, where appropriate empirical therapy increased by 56.0% (p < 0.001). Though smaller, improvements were also seen in complicated cystitis and pyelonephritis. The limited gains in complicated infections may reflect greater clinical complexity, including comorbidities and broader treatment options that complicate strict adherence to guideline-based therapy [27]. 

Clinical outcomes also improved. UTI recurrence rates declined significantly post-AMS-OP (from 25.2% to 11.9%; p = 0.005), and relapses decreased from 8.4% to 3.1% (p = 0.07). Empirical therapy quality improved, with more patients receiving appropriate antibiotic selection, dosage, and duration. Although this study prioritized antibiotic selection as the main outcome, dosage and duration were assessed in relapse cases but not across the full sample, limiting the generalizability of those findings. Future research should incorporate these parameters systematically. The implemented clinical guideline proved to be a useful tool for prescribers, supporting better outcomes through an evidence-based recommendations approach that could be safely adopted across the Latin American region [19]. 

The AMS-OP intervention also led to a more favorable distribution of antibiotic use per the WHO AWaRe classification. After implementation, Access-group antibiotics were prescribed more frequently for OP UTIs overall and for uncomplicated cases in particular (Figure 2a, Figure 2b) [12]. For complicated UTIs, a higher proportion of Watch-group antibiotics is expected. Nonetheless, in patients without risk factors for multidrug-resistant organisms, prescribers should be encouraged to prioritize Access-group agents [18,27]. 

Physician adherence to clinical guidelines improved markedly post-implementation. Regular monitoring of prescribing behavior was instrumental in achieving this, as evidence indicates that physicians are more likely to adjust their prescribing when they know their practices are being tracked [24,28-30]. 

Despite these positive results, sustained training and expansion of stewardship efforts to other infectious conditions, both within and beyond HCB, will be essential to strengthen AMS-OP and promote rational antibiotic use in the broader OP setting. 

Limitations 

This study has several limitations. First, it was conducted in a single private hospital in Costa Rica, which may limit the generalizability of findings to other OP settings nationwide. Nonetheless, given the hospital’s substantial OP volume, similar or even more complex prescribing patterns may exist in other healthcare facilities. 

Second, although treatment duration and dosage were assessed in relapse cases, these parameters were not systematically evaluated across the full UTI cohort. This decision was made to prioritize antibiotic selection as the primary outcome. Future studies should aim to include dosage and duration assessments for a more comprehensive analysis. Lastly, follow-up data may be incomplete, as patients could have sought care at external facilities, limiting the ability to fully track clinical outcomes. 

## Conclusions

The implementation of an AMS-OP at HCB led to significant improvements in optimal antibiotic selection for UTIs, increased physician adherence to clinical guidelines, and better patient outcomes, including reduced recurrence and relapse rates. These results highlight the feasibility and effectiveness of AMS-OP initiatives in promoting rational antibiotic use in Latin American OP settings. 

To ensure long-term impact, integration of AMS-OP into national healthcare strategies, along with ongoing education and monitoring of prescribing practices, is essential. Further research across diverse healthcare environments is recommended to validate and extend these findings, supporting broader adoption of OP stewardship interventions. 
